# A comparative study of the toxic effect of ZIF-8 and ZIF-L on the colonization and decomposition of shaded outdoor mice carrions by arthropods

**DOI:** 10.1038/s41598-022-18322-5

**Published:** 2022-08-20

**Authors:** Fatma El-Zahraa A. Abd El-Aziz, Noha Esmael Ebrahem, Hani Nasser Abdelhamid

**Affiliations:** 1 Zoology Department, Faculty of Science, Assiut University, Assiut, 71516 Egypt; 2 Department of Forensic Medicine and Clinical Toxicology, Faculty of Medicine, Assiut University, Assiut, 71516 Egypt; 3 Advanced Multifunctional Materials Laboratory, Chemistry Department, Assiut University, Assiut, 71516 Egypt; 4 Proteomics Laboratory for Clinical Research and Materials Science, Department of Chemistry, Assiut University, Assiut, Egypt; 5 Nanotechnology Research Centre (NTRC), The British University in Egypt (BUE), Suez Desert Road, El-Sherouk City, Cairo, 11837 Egypt

**Keywords:** Microbiology, Environmental sciences, Materials science

## Abstract

Metal–organic frameworks (MOFs) are promising materials for several applications. Thus, they have been intensively reported and commercialized by several international companies. However, little is known about the fate and risk of MOFs to living organisms. Here, the toxic effect of two Zinc (Zn)-based MOFs; zeolitic imidazolate frameworks (ZIF-8) and leaf-like ZIF (ZIF-L), was tested to investigate the impact of the postmortem period of mice carrions and arthropods which found in decomposing carrions. The data analysis revealed an increase in zinc content over time. Toxicology in forensics studies biological materials for the presence of poisons, such as pharmaceuticals. The toxicology report can provide important details about the types of chemicals present in a person and whether the amount of those substances is in line with a therapeutic dose or exceeds a dangerous level. These findings conclude the possible fate and impact after mortality. This study presents the first study of the toxic effect of ZIFs materials using mice carrions and arthropods (*Sarcophaga* sp. Larvae) via morphological and microscopic studies compared with control, providing important biological information could aid in the environmental impact of the toxic level of MOF materials.

## Introduction

The postmortem period is an essential task for forensic science and environmental concerns^1^. Establishing the cause of death is one of the most critical aspects of a fatality investigation. This feature may be difficult to determine when the corpse is retrieved and decomposed; this feature may be difficult to determine^2^. Carcasses or carrions are natural and vital nutrients for arthropods. So, insects (Phylum: Arthropoda) have considerable importance in forensic^3–5^. Insects could perform as reliable evidence for toxicological analysis when body fluids and tissues are absent or unsuitable for analysis ^6^. These insects will also be nutrients for earth-raising environmental aspects.

The principal aims of forensic toxicology are to institute the presence and detection of toxicants and establish whether these substances give adverse results. Careful exploration of entomological findings affecting the insects dwelling in carrion can aid in discovering a lot of information critical for investigation concerned with the death of an individual that would otherwise be lost in the absence of awareness on the part of forensic science staffs^7^. Decomposition caused by insect activity in and on the body is a continual process that may be quantified, allowing for accurate minimum Postmortem interval (PMI) estimations up to several months after the death, depending on the circumstances. Thus, numerous publications have reported that drugs and toxins can change the development rates of insects feeding on decomposing Carcasses^8^.

Nowadays, forensic science entails using any science to study legal issues. Items, facts, or opinions submitted in a criminal case developed or supported utilizing a body of forensic science commonly employed in criminal trials are forensic evidence. With such a wide variety of arthropods, it’s not surprising that some have evolved to take advantage of the haven we provide or to exploit us and our products. The common Anthropophilic arthropods include cockroaches (Blattodea), silverfish (Thysanura), house flies (Diptera), and house and dust mites (Acari)^9^. These arthropods are not entirely reliant on people. Synanthropic insects such as filth flies, biting midges, punkies, and mosquitoes (Diptera) feed on humans either directly by blood-sucking or indirectly through waste and garbage. Some of these insects have developed an endophilic lifestyle, which means they feed and rest in our homes. Like some stored goods pests, some hematophagous insect species have lost their wild or peridomestic environment and have become utterly reliant on domestic harborage and people. So, Forensic arthropodology is getting much awareness^9^. Sarcophagidae flies are important forensic markers in assessing human decomposition because they can detect a body within minutes of death, even at great distances. This is also linked to human corpses. Miltogrammatinae, Paramacronychiinae, and Sarcophaginae are the three subfamilies of the Sarcophagidae. Over 3100 species are described in this family, divided into 400 genera. Representatives of this family have a worldwide geographic distribution, with most species residing in tropical or mild temperate regions.

Porous materials such as metal–organic frameworks (MOFs), including zeolitic imidazolate frameworks (ZIFs), are the most appealing porous materials in the research and market^10–18^. They were applied for several applications, including gas storage, separation, catalysis, and biomedicine applications^19–28^. They advanced biomedical applications such as drug delivery, phototherapy, and bone/tissue engineering^29–38^. However, little is known about the potential of these materials for health or environmental hazards. Before considering a MOF-based material for biological systems, assessing the material toxicity is necessary. There are currently few relevant studies on ZIF-8 toxicity, and more research is needed in the following areas to evaluate its bioavailability and direct its appropriate applications accurately.

Forensic toxicology is the branch of science that resolves problems in this field of law. Forensic entomotoxicology in death by poisoning investigations has grown in favor. When traditional toxicological samples have decayed or are no longer accessible, insects might be used as an alternative specimen (evidence). Herein, we investigated the toxic effect of ZIF-8 and leaf-like ZIF (ZIF-L) using arthropods as evidence for decomposing carrions. Various analyses have proved the successful detection, identification, and quantification of ZIF-8 and ZIF-L compounds from insects (*Sarcophaga* sp. Larvae) by the number of insects collected from mice carrions of different groups, by morphological characterization using electron microscopies (EM, e.g., scanning (SEM), and transmission (TEM)). To achieve the relationship between combined chemistry and their toxicological impact on arthropods found on carrions, address issues associated with the toxic effects of substances on humans that are germane to legal events. Based on our knowledge, this study is the first time using mice carrions to evaluate the toxic effect of ZIF-8 and ZIF-L.

## Materials and methods

Zn(NO_3_)_2_•6H_2_O, triethylamine (TEA), and 2-methylimidazole (Hmim) were purchased from Sigma-Aldrich (Germany). Stock solutions of Zn(NO_3_)_2_•6H_2_O and  Hmim were prepared by dissolving 25 g and 62.5 g in 100 and 250 mL of H_2_O.

### Synthesis of ZIF-8 and ZIF-L

ZIF-8 and ZIF-L were synthesized at room temperature using water as solvent and Hmim: Zn molar ratio of 25 and 8, respectively^17,19,28,39–42^. Briefly, TEA (0.1 mL) was added to a solution of Zn(NO_3_)_2_•6H_2_O (0.8 mL, 0.84 M) during stirring. Then, a Hmim (8 mL) solution was added, and the solution was stirred for one hour. The materials were then separated using centrifugation and washed using water (2 × 50 mL) and ethanol (2 × 50 mL).

### Study area and Experimental animals

Fifteen male mice Sprague–Dawley mice (aged 21–35 days), with a weight of (20–23 g) average body weight, were used. Mice were delivered from the 4 Animal House, Faculty of Medicine, Assiut University (Egypt). The study was conducted at Assiut University, Faculty of Medicine, from 1 July to 31 August 2021. This location was poor vegetation, and the environment was arid, with typical temperatures of (40 °C / 37 °C: 36 °C / 34 °C). Each mouse was placed on a plate and enclosed by a perforated plastic box to keep birds, cats, and animal customers out. The site's substrate is primarily made up of sand and dust. All methods were performed following Care and Use of Laboratory animals guidelines.

### Drug, dosage, and administration

Mice were employed in this investigation to mimic the decomposition of a human body. Each mouse was placed on an outdoor plate. Mice were classified randomly into three groups; Group I (Control groups): negative control n = 5), received nothing, neither saline nor drugs, rats were anesthetized with 50 mg/kg intraperitoneal thiopental and sacrificed; Group II (n = 5): received double Lethal Dose 50 (LD_50_) of ZIF-8 (2800 mg/kg) once orally by gastric gavage which caused their death; Group III (n = 5): received double LD_50_ of ZIF-L (2800 mg/kg) once orally by gastric gavage which caused their death.

### Arthropods

The carrions were collected daily during the first-month postmortem. Arthropod specimens from the groups were gathered on each visit and transferred to the Laboratory of the Zoology Department, Faculty of Science, Assiut University. Arthropods were put in 72% ethanol or 10% neutral formal into the preserve, and identify the collected arthropods permanently.

### Collection and preparation of samples

From all of the decomposition stages, *Sarcophaga* sp. (flesh fly) were collected by measuring the concentration of zinc, scanning electron microscopy (SEM), transmission electron microscopy (TEM), and ultrastructural studies as shown below:-

### The determination of Zn concentration

The collected *Sarcophaga* sp. were digested in nitric acid (HNO_3_, 70 wt.%, 5 mL) after 1, 2, 3, and 5 days. The solution was sonicated for 30 min in an ultrasonic bath to dissolve *Sarcophaga sp* completely. The residual was removed via filtration to obtain a clear solution for the analysis using Atomic Flame Absorbance (AFA, Buck scientific 210 VGC).

### Scanning electron microscopy (SEM)

*Sarcophaga* sp. Larvae were fixed on cover slides and coated with gold before measurments. Whole Larvaefrom in three groups were selected for 1.5 h in 5 percent glutaraldehyde in sodium cacodylate buffer, washed in distilled water, and dehydrated in ethanol. The drying of the critical spot was completed. Samples were placed on stubs, coated with carbon or gold, and analyzed at 20 kV using a Scanning Electron Microscope (JOEL, JSM 35, Japan).

### Ultrastructural study

*Sarcophaga* sp. Larvae were used to prepare semithin sections before using transmission electron microscopy (TEM, Electron Microscopy Unit, Assiut University). The cross-sections were examined under TEM (JEOL 100-CXII) operated at 80 kV. The results were presented as micrographs.

### Statistical analysis

Data represented observed numbers of Arthropods stage. Data obtained were subjected to statistical analysis of variance (ANOVA) test using Open Epi version 2.3.1. A *P*-value of ≤ 0.05 was accepted as statistically significant.

### Statement of ethics

This study was approved by the Ethics Committee affiliated with Assiut University Hospital. All procedures performed in the study involving animal participants were following ARRIVE guidelines.

### Ethical approval and consent to participate

Protocols approved by the Laboratory Animal Care and Use Committee of the Affiliated Assiut University and Faculty of Medicine.

## Results and discussion

### Materials characterization

**Figure 1:**
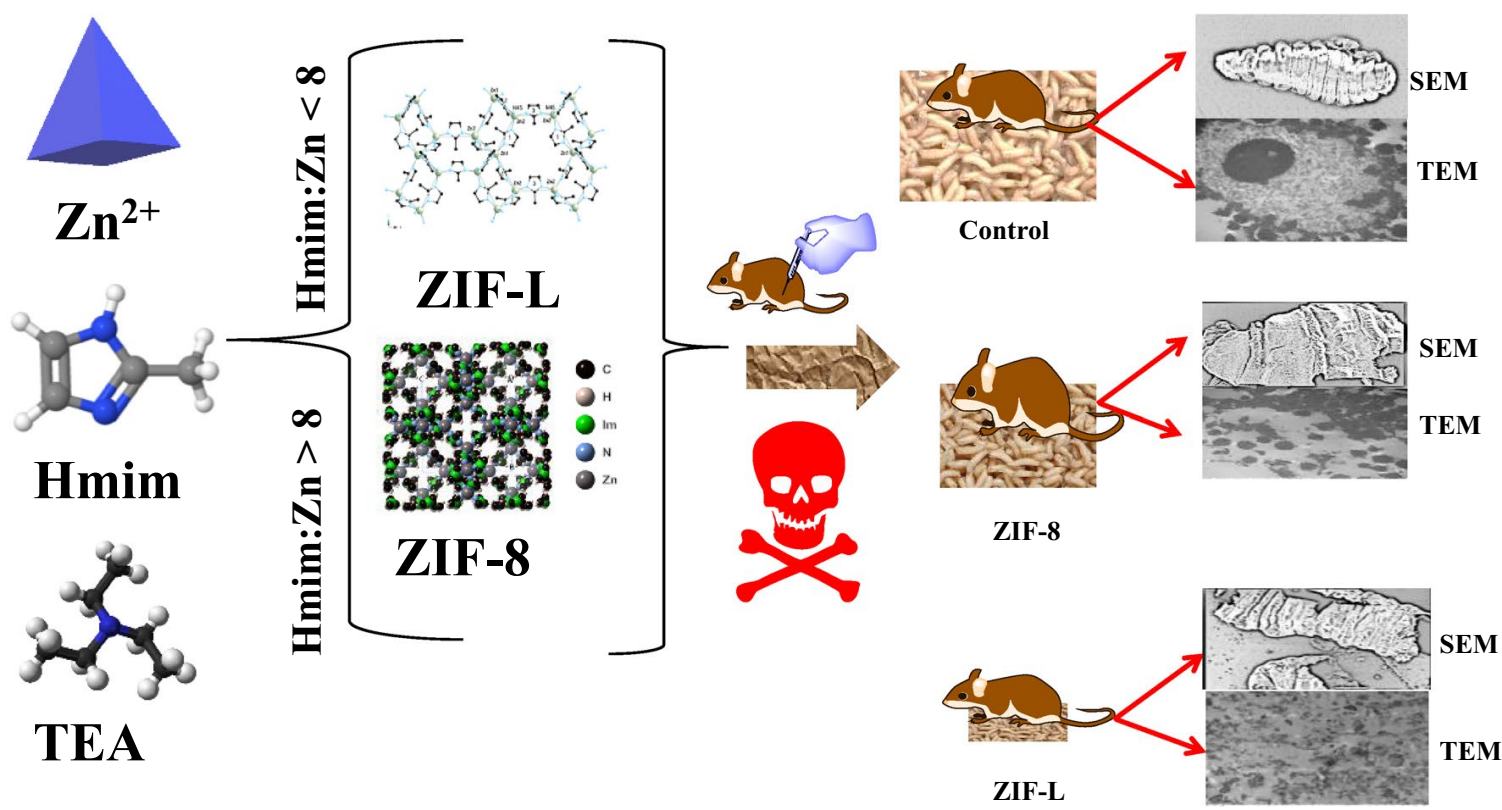
Synthesis of ZIF-8 and ZIF-L and their applications for cytotoxicity.

**Figure 2:**
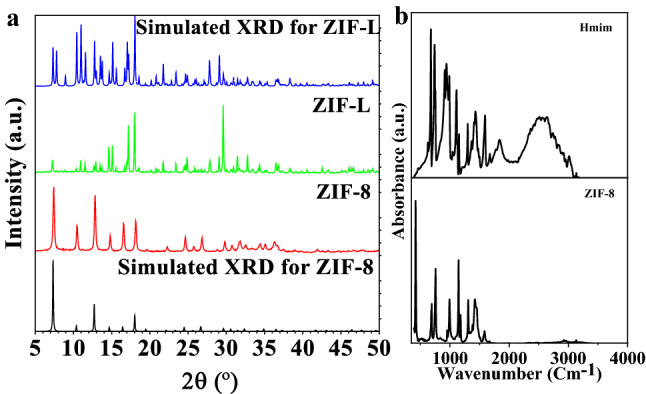
Characterization of ZIF-8 and ZIF-L using (**a**) XRD and (**b**) FT-IR spectra.

**Figure 3:**
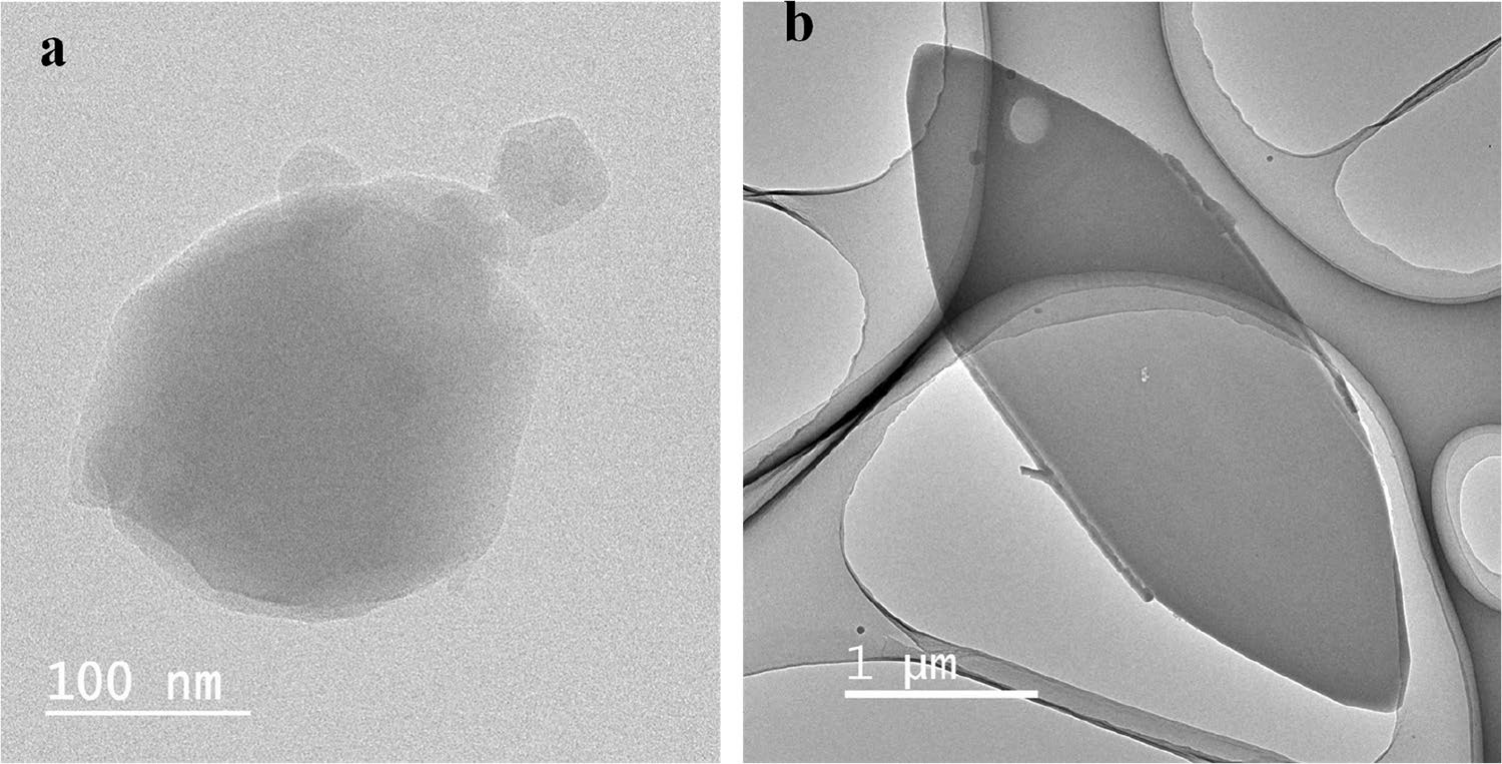
TEM images of (**a**) ZIF-8 and (**b**) ZIF-L.

The schematic representation for the synthesis of ZIF-8 and ZIF-L is presented in Fig. 1. The materials consist of zinc metal nodes and Hmim as a linker. Two different isomers of ZIF-8 and ZIF-L can be obtained with chemical formulas of Zn(mim)_2_ and Zn(mim)_2_•(Hmim)_1/2_•(H_2_O)_3/2_, respectively. The synthesized materials were characterized using XRD (Fig. 2a), FT-IR (Fig. 2b), and TEM images (Fig. 3).

XRD patterns for ZIF-8 and ZIF-L reveal the successful synthesis of pure phases of ZIF-8 and ZIF-L materials (Fig. 2a). They show high and robust diffraction signals indicating high purity. The FT-IR spectra confirm the formation of ZIF materials. The FT-IR spectra for ZIF-8 and Hmim are shown in Fig. 2b. The bands in the wavelength region of 500–1350 cm^-1^ and 1350–1500 cm^-1^ are assigned to the bending and stretching of the imidazolate ring, respectively. After forming ZIFs materials, there is no signal referring to the N–H bending and the stretching vibrations of the free Hmim at 1678 cm^-1^ and 1581 cm^-1^, indicating the absence of a free linker in the final products. A new peak around 420 cm^-1^ corresponds to Zn-N stretching (mim). The particle size and morphology were determined using TEM images (Fig. 3). TEM images show cubic and leaf-like particles for ZIF-8 and ZIF-L particles with particle sizes of 25–100 nm and 10 µm, respectively (Fig. 3). Data analysis confirms the formation of the pure phase of ZIF-8 and ZIF-L.

### The mice carrions decomposition Pattern and arthropods fauna of the decomposition stages

**Figure 4:**
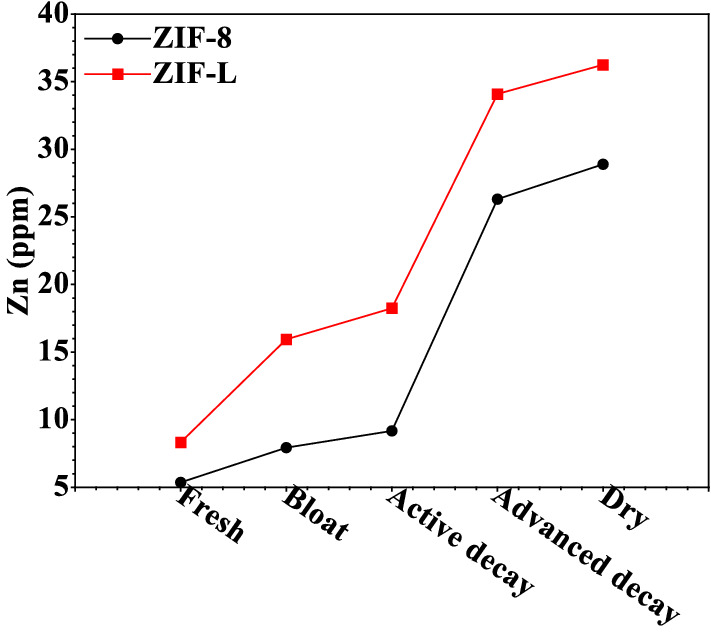
Zinc concentration in larvae collected from the carrions treated with ZIF-8 and ZIF-L.

The first and vital insects species to visit the decomposing carrion in this investigation was *Sarcophaga* sp. (flesh fly), all the decomposition period was 70 days, *Sarcophaga* sp. (flesh fly) were collected from decomposition stages (the fresh stage, the bloated stage, the active stage, the advanced decay stage and dry decay stages) (Fig. 4).

ZIF-8 nanoparticles (NPs) have a wide range of cytotoxic effects. Initially, ZIF-8 NPs have a higher bioavailability than typical zinc-containing nanoparticles such as ZnO NPs, allowing more cells to absorb more zinc. The elevated zinc concentration may then inhibit the GR enzyme, reducing the amount of GSH generated by the cells. ZIF-8 NPs will also raise the level of cellular ROS. GSH depletion would raise the level of ROS in the cells even more. As a result, the cell's inflammatory response will rise, causing inflammation-related genes such as CCL4 and IL6 to be upregulated. Cell necrosis will occur as a result. The concentration of Zn inside the *Sarcophaga* sp. (flesh fly) larvae of the different stages of decomposition was reported, as shown in Fig. 4. The Zn level within the larvae collected from the carrions in group III (for ZIF-L) is higher than that of the larvae collected from the larvae collected carrions in group II (for ZIF-8). The data analysis results also clarified an ascending increase in the concentration of Zn from the fresh stage to The dry decay stages (Fig. 4).

**Figure 5:**
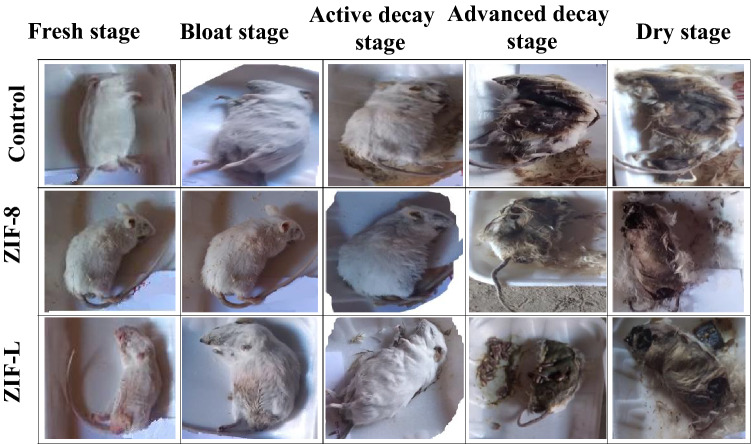
Decomposition of the postmortem interval (PMI) for mice carrions showing the decomposition stages, Group (1); Control, Group (2); ZIF-8 toxicity and Group (3); ZIF-L toxicity.

At the fresh stage, a group I, there were only five families and six species of arthropods without the appearance of pupa or larva 2. While in group II, there were only three families and 3species without the appearance of pupa and larva 2, and in group III, there were only four families and 4species without pupa formation (Table 1 and Fig. 5).

**Table 1:** Comparison between the studied groups in Arthropods of Forensic during 0-7 days.

Variables		Group (I)		Group (II)		Group (III)	
		No	%	No	%	No	%
Fresh stage(0-1 day)	Eggs	8	72.7	3	33.3	6	50
	Larva 1	2	18.3	2	22.2	1	8.3
	Larva 2	0	0	0	0	1	8.3
	Adult	1	9	4	44.4	4	33.3
	Total	11		9		12	
Bloated stage(2–4 Days)	Eggs	26	13.1	12	15.6	2	1.7
	Larva 1	48	24.2	11	14.3	31	27
	Larva 2	29	14.6	14	18	68	59
	Larva 3	39	19.7	5	6.5	5	4.3
	Adult	56	28.3	5	6.5	9	7.8
	Total	198		77		115	
Active stage(5-7 days)	Eggs	27	7.6	27	8.7	30	6.5
	Larva 1	80	22	88	28.2	121	26
	Larva 2	57	17	108	34.6	193	41.5
	Larva 3	31	9.3	31	9.9	66	14.2
	Pupae	77	21	47	15.1	47	10.1
	Adult	83	25	8	2.6	8	1.7
	Total	355		312		465

The carrions in the bloated stage were normally bloated and had a rotten stench, with decomposition fluids pouring beneath the corpse. Group II is more than the other two groups. *Chrysomya albiceps*, *Musca domestica*, and *Sarcophaga* sp., were the first invaders. Numerous isopods, spiders, and beetles were found beneath the carrions. Group II showed less number of eggs and larva than other groups without the appearance of pupa in all groups (Table 1 and Fig. 5).

At the active stage, Skin fur separation, all body surface intact, and larval infestation are found in group I, whereas dry body liquefied tissues in dryness form arborization were seen in groups II and III in addition to a minor larval infestation. Group III showed more eggs and larvae than others (Table 1).

At the advanced decay stage, odors were lower in all groups. Carrions were severely dehydrated and had lost a significant amount of weight. The bodies were starting to detach from the dry skin. While the internal systems become available to feed arthropods on them, this will cause an increase in the rate of Zn within the larvae. Several insects, isopods, and beetles were discovered. Group III showed more larvae than other groups (Table 2 and Fig. 5).

**Table 2:** Comparison between the studied groups in Arthropods of Forensic during 8–30 days.

Variables		Group (I)		Group (II)		Group (III)	
		No	%	No	%	No	%
Advanced decay stage(8-9 day)	Eggs	33	20.3	33	18.5	27	7.7
	Larva 1	20	12.3	40	22.4	157	44.7
	Larva 2	31	19.1	53	29.8	117	33.3
	Larva 3	17	10.5	17	9.6	17	4.8
	Pupae	27	16.7	28	15.7	28	8
	Adult	34	21	7	3.9	5	1.4
	Total	162		178		351	
Dry decay stage(10–30 day)	Eggs	21	7	21	8.5	20	6
	Larva 1	50	16.7	51	20.6	84	25.4
	Larva 2	22	7.4	47	19	61	18.4
	Larva 3	60	20	60	24.3	85	25.7
	Pupae	61	20.4	63	25.5	81	24.5
	Adult	85	28.4	5	2	2	0.6
	Total	299		247		331	

The soft tissue vanished in all groups at the dry decay stages, and the carrion odor began to dissipate. Dry skin and bone made up the mice. Calliphoridae, Muscidae, and Sarcophagidae had fewer specimens collected. Group III revealed more larva and pupa than other groups (Table 2 and Fig. 5).

- Dermestes Frisch, Dermestes maculates, and Muscadomestica were significantly prominent in Group I. Sarcophaga sp., Muscadomestica, and Dermestes maculates were quite apparent in group II. While Sarcophaga sp., Muscadomestica, and Wohlfahrtia Magnifica were significantly prominent in group III (Table 3 and Fig. 5).

**Table 3:** Comparison between isolated species of Arthropods in the studied groups during the whole period of the study (from 0 to 31 days).

Family	Species	Group I		Group II		Group III		Total	P- value*
		No	%	No	%	No	%		
Calliphoridae	Chrysomya albiceps (blow fly)	69	28.4	69	28.4	75	43.2	213	0.3
Apidae	*Apis sp.*	6	28.5	1	4.8	14	66.7	21	< 0.05*
Muscidae	*Musca domestica* (house fly)	220	32.6	175	26	280	41.5	675	< 0.05*
Sarcophagidae	*Sarcophaga* sp. (flesh fly)	180	31.9	189	33.5	287	50.8	565	< 0.05*
	Wohlfahrtia magnifica	68	28.7	75	31.6	176	74.3	237	< 0.05*
	Parasarcophaga orgyrostama	23	27.4	18	21.4	43	51.2	84	< 0.05*
Dermestidae	Dermestes maculates (hide beetle)	168	42.7	95	24.3	130	33	393	< 0.05*
	Dermestes frischi	197	47.5	70	16.7	148	35.7	415	< 0.05*
Histeridae	Saprinus sp. ( clown beetles)	31	54.4	19	33.3	17	29.8	57	0.7
Pteromalidae	Nasonia sp.	24	61.5	9	23	6	15.5	39	0.8
Lycosidae	Spider	20	28.2	26	36.6	25	35.2	71	0.4
Pyroglyphidae	Dermatophagoides sp. ( dust mites)	4	66.7	2	33.3	0	0	6	NA
Cimicidae	Cimex lectularis ( Bed bugs )	9	81.8	2	18.2	0	0	11	NA
Porcellionidae	Porcellio laevis (woodlouse )	4	80	1	20	0	0	5	NA

### Morphological and microscopic studies of Sarcophaga sp. Larvae

**Figure 6:**
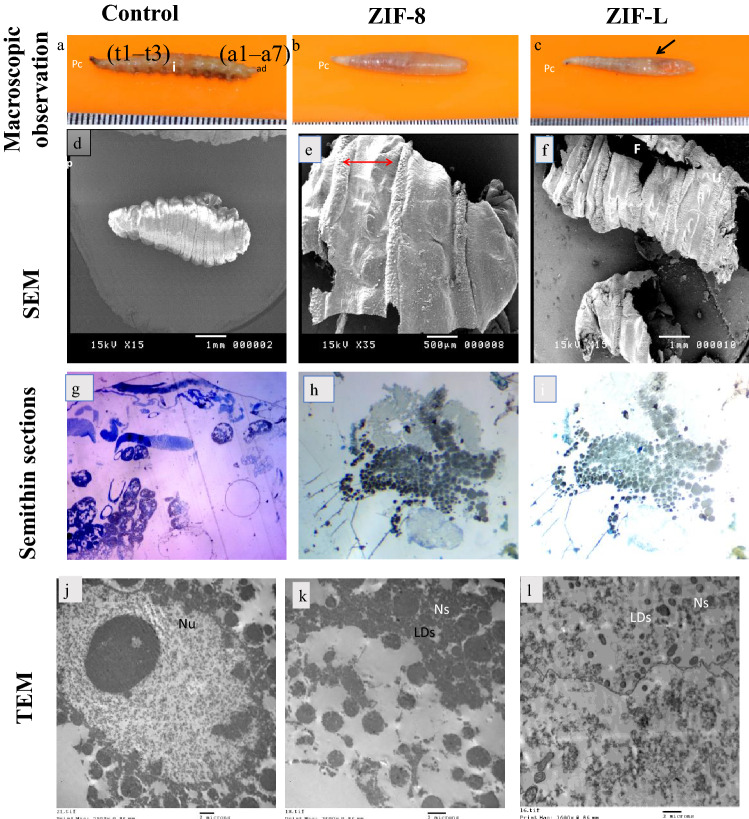
Photographs, showing (**a**) larvae of *Sarcophaga* sp., (**b**) from group: a, group (1) control, Larvae elongated, truncated posteriorly and tapering toward the anterior extreme curved downward, they are cylindrical, segmented. The body is divided into pseudocephalon (pc), three thoracic segments (t1–t3), seven abdominal segments (a1–a7) and an anal division (ad), which carries the posterior spiracle, intestine (i), (**c**) from group (2) Larvae were normally bloated, had a rotten stench, and d: from group (3) Larvae were completely bloated, a strong putrid odor with fluids oozing and the presence of abscess (row). Scanning electron micrographs showing: (**a**) The whole body, (**d**) group (1) control, Showing ventral view showing pseudocephalon (PC), anterior spiracles (as) the posterior spiracles (Ps.), well developed annular bands (ab) of intersegmented spines, is in 4–6 rows of pinted spines, the grooves of the body are obvious, (**e**) group (2) larva showing broken with hyperplasia of the skin epithelial layer, (**f**) group (3) larva showing broken, dryness of the skin with formation of ulcer(u)and (**k**) the presence of fissure (F) .Photomicrographs of semithin sections of the frontal part *Sarcophaga* sp. Larvae, (**g**) group (1), (**h**) group (2) and (**i**) group (3). Transmission electron microscopy (TEM) micrographs of anterior part of *Sarcophaga* sp. Larvae, (**j**) group (1), (**k**) group (2) and (**l**) group(3).nucleus (Nu) , mitochondria (M), numerous lipid droplets (LDs), naopartecticles (Ns).

In Group I(control), The body is divided into pseudocephalon (pc), three thoracic segments (t1–t3), seven abdominal segments (a1–a7), and an anal division (ad), which carries the posterior spiracle (ps) intestine (i), *Sarcophaga* sp. larvae have two pairs of spiracles (anterior and posterior), and there is no visible demarcation between the thorax and the abdomen. There is a change in the elongated, truncated posteriorly, and tapering toward the anterior extreme curved downward; they are cylindrical, segmented (Fig. 6a). In group II, Larvae were normally bloated, and had a rotten stench (Fig. 6b), and in group III. The larvae were inflated, with a strong putrid stench, leaking fluids, and an abscess (Fig. 6c).

For scanning electron micrographs showing: Group I, the whole body, ventral view showing pseudocephalon (PC), anterior spiracles (as) the posterior spiracles (Ps.), well developed annular bands (ab) of intersegment spines (s) is in 4–6 rows of pinted spines, the grooves of the body are apparent, anterior portion showing three thoracic segments with anterior spiracular, maxillary palpus consists of several sensillae, In the frontal and inner margins, a group of small spines delimiting the atrial cavity, the array (R) of the cuticular ridges (CR) were found associated between the mouth (V-shape dorsal grooves) and the prominent hooks. (cs) Cephalo-pharyngeal skeleton., anal segment, the posterior spiracles are enclosed within the deep stigmas cavity, the spiracular openings (caves), pores, perispiracular glands, and sensilla, rima, and rays are also visible, and the fleshy projections (fp) (Fig. 6d). Group II, larvae showing broken with hyperplasia of the epithelial layer, presence of fissure and broken with pores (Fig. 6e) and Group III, larva showing broken, dryness of the skin with formation of an ulcer, accumulation of crusts and presence of fissure (Fig. 6f). Photomicrograph of a semithin section of Larvae of the control group revealed typical architecture, and Large, prominent nuclei (Nu) were also typical (Fig. 6g). Semithin section of Groups (II and III) fat body cells. Lipid droplets (LD) of variable sizes and densities were evident throughout the cytoplasm (Fig. 6h-i). Ultrastructural evidence for induction of apoptosis or any other form of cell death in body cells of Groups (II and III) by toxicity, but in group III the toxicity is higher than in group II (Fig. 6j-l).

In this study, various arthropods were collected on the mice carrions belonging to eleven orders; and have been implicated by other researchers of forensic^43,44^. These species of arthropods are significant in estimating the time of death. The second most critical variable determining the decomposition of a cadaver after the temperature is access for arthropods to the body. The current study investigated the level of Zn that was increased in the decomposition stages from the fresh stage, the bloated stage, the active stage, the advanced decay stage, and the dry decay stage. Moreover, observation by photographs, SEM, semithin, and TEM of Sarcophagasp. (flesh fly) larvae. The groups that received the ZIF-8 and ZIF-L received apparent damage as visible on TEM micrographs. These findings are also consistent with our recent studies, which reveal that this ZIF-8 and ZIF-L toxin causes many cell death in vitro, with apoptosis being the most common death mechanism activated.

Emeka et al. worked on three pig carrions for 60 days; arthropods were gathered twice a day for the first week and once daily for subsequent weeks^45^. Five decomposition phases were identified; the dry decay stage took the longest to decompose, the advanced decay stage, and the fresh stage took the shortest. This result follows the current development.

Morphological Sarcophaga sp. (flesh fly) larvae damage index was used for evaluation of the degree of larvae damage; it depends mainly on the percentage of ZIF-8 and ZIF-L in the tail, so it is considered an accurate measure for the degree of toxin when we compare with normal as reported by Szpila et al. (2015).

Salim et al. established potential underestimation after death based on the damaged interpretation of the development of C. albiceps larvae reared on the tissues of rabbits carrionss containing morphine^46^. Conversely, work by Kharbouche et al. revealed an increased rate of development for larvae of Lucilia sericata and Boettchersica peregrine reared on minced pig liver containing different codeine concentrations^47^. Thus, the same species could react differently toward two molecules belonging to the same family. Keshavarzi et al. pointed out that insect succession became comparable among the dealt with and untreated rabbit carcasses. However, the styles of the sequence of Chrysomya albiceps and Calliphora vicina differed barely among dealt with and untreated carcasses.

The present study discovered that larvae reared on decomposing tissue-containing ZIF-L had retardation of development compared with the ZIF-8 groups and control group as the means of weights, lengths significantly, and widths of the ZIF-L and ZIF-8 groups were considerably lower than those of the control larvae at different time intervals.

In the present study, Sarcophagasp. (flesh fly) larvae were of great importance in forensic medicine, as they appeared in the five stages of decomposition, so we used the flesh fly larvae to estimate zinc inside the larvae. On the other hand, study the morphological, SEM, and TEM electronic microscopy to review the changes that occurred in control and treated groups because it is substantial evidence in forensic medicine. This investigation presented for the first time the use the morphological changes, electronic microscopy studies, and Zn estimate of arthropods as evidence of toxins that are used in suicide or death, even by mistake.

## Conclusions

The decomposition of carcasses and carrions arthropods (larvae of Sarcophaga sp.) significantly changes terrestrial ecosystems. The present work has shown that a careful study of two chemical compounds, ZIF-8 and ZIF-L, and their interaction with the decomposition of carcasses and entomology has profound effects on the arthropods (larvae) Sarcophaga sp.) forensically significant invertebrates. Our study reveals that ZIF-8 and ZIF-L can be present in the investigated anthropods feed on mice carriers. It will aid in improving the evidential usefulness of entomology to the Forensic Science and Policing communities in criminal investigations. Thus, further research is highly required in the future to determine the factors that govern the toxicology and the changes in the soil content after the decomposition of carrion anthropods.

## Data Availability

The data supporting this study's findings are available from the corresponding authors upon reasonable request.
